# Cyclophilin A stabilizes the HIV-1 capsid through a novel non-canonical binding site

**DOI:** 10.1038/ncomms10714

**Published:** 2016-03-04

**Authors:** Chuang Liu, Juan R. Perilla, Jiying Ning, Manman Lu, Guangjin Hou, Ruben Ramalho, Benjamin A. Himes, Gongpu Zhao, Gregory J. Bedwell, In-Ja Byeon, Jinwoo Ahn, Angela M. Gronenborn, Peter E. Prevelige, Itay Rousso, Christopher Aiken, Tatyana Polenova, Klaus Schulten, Peijun Zhang

**Affiliations:** 1 Department of Structural Biology, University of Pittsburgh School of Medicine, Pittsburgh, Pennsylvania 15260, USA; 2 Pittsburgh Center for HIV Protein Interactions, University of Pittsburgh School of Medicine, Pittsburgh, Pennsylvania 15260, USA; 3 Department of Physics and Beckman Institute, University of Illinois at Urbana-Champaign, Urbana, Illinois 61801, USA; 4 Department of Chemistry and Biochemistry, University of Delaware, Newark, Delaware 19716, USA; 5 Department of Physiology and Cell Biology, Ben-Gurion University of the Negev, Be'er-Sheva, 84105, Israel; 6 Department of Microbiology, University of Alabama at Birmingham, Birmingham Alabama 35294, USA; 7 Department of Pathology, Microbiology and Immunology, Vanderbilt University School of Medicine, Nashville, Tennessee 37232, USA

## Abstract

The host cell factor cyclophilin A (CypA) interacts directly with the HIV-1 capsid and regulates viral infectivity. Although the crystal structure of CypA in complex with the N-terminal domain of the HIV-1 capsid protein (CA) has been known for nearly two decades, how CypA interacts with the viral capsid and modulates HIV-1 infectivity remains unclear. We determined the cryoEM structure of CypA in complex with the assembled HIV-1 capsid at 8-Å resolution. The structure exhibits a distinct CypA-binding pattern in which CypA selectively bridges the two CA hexamers along the direction of highest curvature. EM-guided all-atom molecular dynamics simulations and solid-state NMR further reveal that the CypA-binding pattern is achieved by single-CypA molecules simultaneously interacting with two CA subunits, in different hexamers, through a previously uncharacterized non-canonical interface. These results provide new insights into how CypA stabilizes the HIV-1 capsid and is recruited to facilitate HIV-1 infection.

## Abstract (web summary)

The host cell factor cyclophilin A (CypA) interacts with the HIV-1 capsid and regulates infectivity. Here the authors combine cryo-EM, solid-state NMR and all-atom MD simulations, identifying an interaction interface between CypA and the HIV capsid that stabilizes the viral capsid and regulates infectivity.

## Graphical abstract

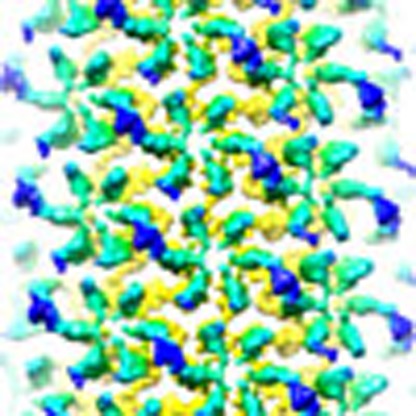

Infection by retroviruses, including HIV-1, is critically dependent on the functions of the viral capsid^1^,^2^,^3^, which plays multiple roles during replication, including the prevention of innate sensor triggering^4^,^5^ and regulation of reverse transcription^1^,^6^ and nuclear import^7^,^8^,^9^. Such functions are highly dependent on interactions between the viral capsid and cellular factors. The host cell protein cyclophilin A (CypA) binds directly to the HIV-1 capsid and modulates capsid uncoating and viral infectivity^10^,^11^,^12^. Interference with CypA-binding inhibits HIV-1 replication in cell culture^13^,^14^,^15^. In addition, host cell proteins containing the CypA domain, including Trim-Cyp and Nup358, interact directly with the viral capsid and control HIV-1 infection^8^,^16^,^17^.

CypA-binding appears to stabilize or destabilize the HIV-1 capsid, depending on the cell type^6^,^18^. In addition, the interaction between CypA and HIV-1 CA promotes HIV-1 infection of human cells^19^,^20^, yet, in non-human primates, the same interaction enhances the anti-HIV-1 restriction activity of Trim5α (refs 21, 22, 23). Furthermore, while CypA promotes HIV-1 reverse transcription in human cells, its cell-type specific effect is best correlated with nuclear entry, and probably involves an unknown CypA-dependent host restriction factor^24^. Owing to this complexity, understanding the role of CypA in HIV-1 infection has been difficult, especially given the limited structural information that is available.

The crystal structure of CypA in complex with the CA N-terminal domain (CA_NTD_) shows that CA residues A88-G89-P90 and the configuration of the CypA-binding loop are important for the binding interaction^11^. Binding of CypA to the CypA-binding loop of the viral capsid is essential for viral infectivity^25^,^26^, as substitutions in loop residues G89 or P90 in HIV-1 CA are deleterious to replication^14^,^21^,^27^. CypA binding to monomeric CA can be detected at high protein concentration^11^, but binding is enhanced by CA multimerization^10^, suggesting that CypA favours binding to an assembled capsid. Despite extensive studies on the interaction between CypA and HIV-1 CA, structural information on how CypA interacts with the assembled viral capsid, and an understanding of the consequences of the interaction for capsid function, are lacking.

To elucidate the molecular interactions between CypA and the HIV-1 capsid, we determined the structure of CypA in complex with an HIV-1 CA tubular assembly at 8-Å resolution by cryoEM. The density map, combined with structure-guided molecular dynamics (MD) simulations, unexpectedly revealed a novel, non-canonical, second capsid-binding site on CypA that is vital for stabilizing the viral capsid. The finding was confirmed experimentally by solid-state NMR.

## Results

### Binding of CypA to wild-type (wt) CA assemblies

**Figure 1:**
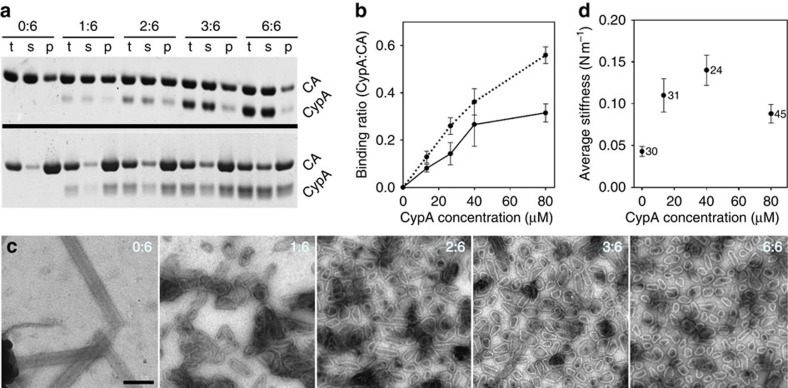
Interaction of CypA with HIV-1 CA assemblies. (**a**) Binding of CypA to pre-assembled wt CA tubes in 1 M NaCl (top) and co-assembly of CypA and CA in 2.25 M NaCl (bottom); the molar ratio of CypA:CA in each condition is indicated. Reaction products before centrifugation (t) and after centrifugation, supernatant (s) and pellet (p), were analysed by 4–12% gradient gel. (**b**) The binding ratios of CypA:CA were quantified for the pre-assembled sample (solid line) and for the co-assembled sample (dotted line), calibrated using the input ratio as standard. (**c**) EM images of negative stained samples of CypA-CA complexes co-assembled in 2.25 M NaCl with the indicated CypA:CA molar ratios. Scale bar, 200 nm. (**d**) AFM measurements of the stiffness of CA assemblies in the presence of CypA at CypA:CA molar ratios of 0:6, 1:6, 3:6 and 6:6. At least 24 particles (numbers shown to the right of each data point), obtained from 2–3 individual assembly reactions, were measured for each stiffness value. Error bars represent the s.e.m.

We began our studies by characterizing the binding of CypA to HIV-1 CA tubular assemblies at various CypA:CA molar ratios. Binding was measured in a high-speed centrifugation assay, after incubation of CypA with a fixed concentration of pre-assembled wild-type (wt) CA tubes. Incubation of CypA with CA tubes, up to a 6:6 molar ratio, resulted in co-sedimentation of CypA and CA (Fig. 1a, top), with the CypA:CA binding ratio increasing as the CypA concentration increased, up to 40 μM, beyond which point, binding was saturated (Fig. 1b, solid line). Interestingly, while a relatively low amount of CypA (CypA:CA input ratios ≤ 2:6) slightly enhanced the amount of CA in the pelleted fraction (Fig. 1a top), CypA:CA ratios above 2:6 led to a reduction in the amount of pelleted CA (Fig. 1a, top). This finding is consistent with an earlier report that high molar ratios of CypA:CA alter CA assembly *in vitro*^28^.

We also tested the effect of CypA on the process of CA assembly by including it in the CA assembly reaction under the high-salt condition (2.25 M NaCl) (Fig. 1a, bottom). In this case, CypA binding was consistently higher compared with when it was added to pre-assembled CA tubes (Fig. 1b, dotted lines). However, the presence of CypA during CA assembly induced marked morphological changes in the resulting CA assemblies (Fig. 1c): in the absence of CypA, long CA tubes were formed; at an intermediate level of CypA, short tubes and cones were generated; and, at high CypA concentrations (6:6 CypA:CA molar ratio input), very small cones and spheres were observed, indicating a significant increase in the curvature of the assemblies. These data suggest that CypA influences the curvature of CA assemblies possibly by preferentially binding to and stabilizing CA assembly conformations with a high degree of curvature, a distinctive feature that had not been previously appreciated but can be clearly explained by the structure presented here (further sections). This influence of CypA on the capsid curvature may shed new light on the maturation process, since CypA is packaged into viral particles and may facilitate the formation of cones by stablizing a high degree of curvature at the ends during maturation. Similar to the pre-assembled tubes, CA was reduced in the pelleted fraction with higher CypA:CA molar ratios in the co-assembly reaction (Fig. 1a).

### CypA affects the stiffness of CA assemblies

To determine the effect of CypA on CA stability, we used atomic force microscopy (AFM) operated in the nano-indentation mode to measure the stiffness of wt CA assemblies as a function of CypA level. The point stiffness measured for CA without CypA was 0.043±0.006 N m^−1^ (*n*=30) (Fig. 1d). A gradual elevation in CypA level resulted in an increase in CA stiffness (1:6 CypA:CA ratio is 0.11±0.02 N m^−1^ (*n*=31) and 3:6 is 0.14±0.018 N m^−1^ (*n*=24)). The stiffness value represents the amount of force that is required to deform the CA structure or, alternatively, the ability of the structure to withstand an external force. In the case of a CA assembly, this ability is governed by the interaction strength between its building blocks, namely, CA hexamers. Thus, higher stiffness values represent stronger interactions between the building blocks of the CA lattice. Incorporation of CypA into the CA structure increased the effective interaction strength between the CA hexamers. This effect was maximal at a 3:6 CypA:CA ratio. Interestingly, further addition of CypA to a 6:6 ratio reduced the stiffness of CA by almost twofold (0.088±0.011 N m^−1^ (*n*=45)), compared with the stiffness obtained at a 3:6 ratio. However, the CA stiffness at 6:6 remains significantly larger than CA lacking CypA. These results suggest that CypA incorporation in the capsid structure strengthens the stability of capsid in a stoichiometrically dependent manner, with the efficiency of this effect being notably decreased at high (6:6) CypA to CA molar ratios.

### CryoEM structure of CypA-CA tubular complex

**Figure 2:**
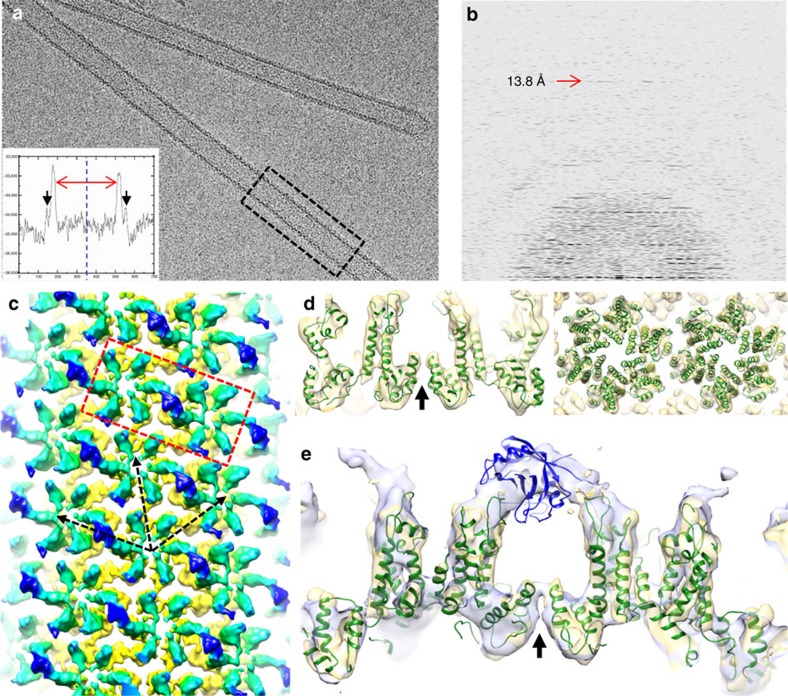
CryoEM reconstruction of CypA /CA tubular assemblies. (**a**) CryoEM image of a CypA-CA tubular assembly at 2:6 CypA:CA input ratio. Inset, radial density profile from the boxed region showing the CypA density (black arrows) decorating the CA tubular assembly (red arrows). (**b**) Fourier transform of a CypA-CA tubular assembly. Arrow indicates a layer line at 13.8 Å resolution. (**c**) 3D density map of the CypA-CA assembly complex reconstructed at 8 Å resolution (see Supplementary Fig. 1). The density (contoured at 2σ) is coloured radially from yellow-green (CA) to blue (CypA). CypA selectively binds to the CA array that has the highest curvature. (**d**) Rigid body docking of CA hexamers (3J4F, green ribbon) into the CA region of the density map (yellow density), viewed from the side and top. The area shown is from the boxed region in C. (**e**) Aligning the CypA-CA complex (PDB: 1AK4) to the docked CA hexamer model, overlaid with the density maps contoured at 2σ (yellow) and at 1.5σ (blue). The CypA molecule (blue ribbon) is situated right at the bridging density, across two CA hexamers (green ribbons) and above the CA dimer interface (arrow).

To determine the structure of the CypA-CA tubular assembly complex, we chose the CypA-CA ratio and assembly conditions that yielded homogeneous complexes suitable for cryoEM: pre-assembled CA tubes with CypA added at 2:6 CypA:CA molar ratio (Fig. 1a top). Addition of CypA resulted in well-separated, long helical tubes with a layer of density decorating the CA tube surface (Fig. 2a, inset, black arrows). Such tubes are well-ordered to at least 13.8-Å resolution, as indicated by the Fourier transform (Fig. 2b). We carried out three-dimensional (3D) reconstruction of the CypA-CA tubular complex using the iterative real-space helical reconstruction method^29^. Combining 19 high-resolution tubes belonging to a single helical symmetry of (−8, 13) (see ref. 29 for information regarding helical symmetry), we determined the cryoEM structure of the CypA-CA tubular complex at 8-Å resolution, as indicated by the Fourier shell correlation of 0.5 (Supplementary Fig. 1).

**Figure 3:**
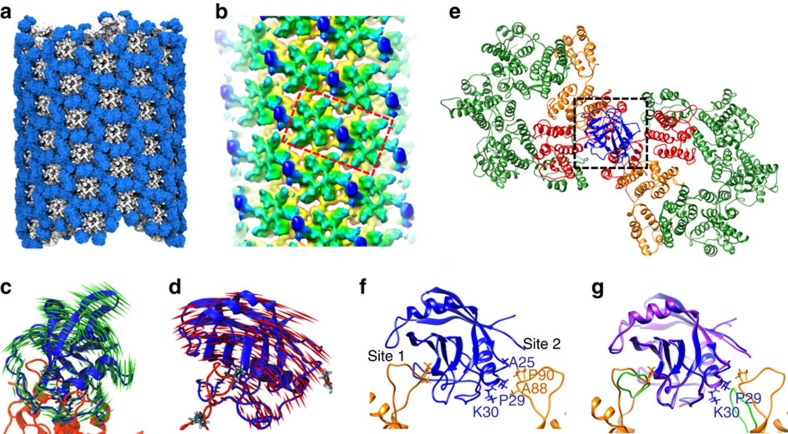
All-atom MD simulations of the CypA-CA complex in a helical assembly. (**a**) A fully saturated CypA-CA tubular complex model with extensive clashes between CypA and CA and between adjacent CypA molecules. CypA is in blue and CA in grey. (**b**) A density map averaged from ten helically symmetrized MD models of CypA-CA tubes. Colour scheme is the same as that in Fig 2c. The density is contoured at 2σ. The red box encloses the two CA hexamers in complex with CypA that are shown in **e**. (**c**,**d**) Porcupine representation of the most dominant mode from principal component analysis performed on the trajectories of the bridging (**c**) and non-bridging (**d**) binding modes of CypA (blue). (**e**) Result from an all-atom MD simulation of the CypA-CA complex comprising two CA molecules (orange) and one CypA (blue) within a tubular configuration, shown with two CA hexamers (green). The CA molecules making the dimer interface are in red. (**f**) An enlarged view of the CypA-CA complex from the boxed region in E rotated 90°, illustrating the two CA binding sites on CypA: site 1 is the canonical binding site and site 2 is the non-canonical binding site (see text). Selective residues at the site 2 interface are labelled. (**g**) Overlay of the MD CypA-CA model with the crystal structure of the CypA-CsA complex (PDB: 2RMA, CypA in purple and CsA in green), illustrating that CypA interacts with a second CsA at site 2.

The resulting density map displays a number of intriguing features. First, among the three CA helical arrays in the assembly (Fig. 2c; dashed black arrows denote the three directions along which helical tubular arrays are formed, each direction corresponding to a different curvature), CypA binds preferentially along the most highly curved array (Fig. 2c, Supplementary Fig. 2a). Very little CypA binds to the CA arrays that run in the other two helical directions. The same binding preference was observed in an independent reconstruction generated from tubes with a very different helical symmetry (−12, 11) (Supplementary Fig. 2b). Such selective binding explains the sub-stoichiometric binding ratios observed in our CypA-binding assays (Fig. 1b). Second, while the CA region is highly ordered and all the α-helices are well resolved (Fig. 2d, Supplementary Fig. 1b,c), the resolution at the CypA region is poorer (Fig. 2e, Supplementary Fig. 1b,c). This could be because of the known flexibility of the CypA-binding loop^11^,^30^, which would give rise to variable CypA orientations, and/or to partial occupancy by the CypA molecule. Indeed, when individual images were aligned based on the CA density (Supplementary Fig. 1a), the resolution for the CA region was improved, but the density corresponding to the CypA molecule became less well-defined (Supplementary Fig. 1b,c). Third, rigid body docking of the hexamer model (PDB: 3J4F) derived from our previous study^30^ showed a near-perfect fit into the CA density (Fig. 2d), with a cross-correlation coefficient (CCC) of 0.84 between the atomic models of the hexamer and the density map, suggesting that CypA binding to a pre-assembled tube has little effect on the overall structure of the CA hexamer. Finally, aligning the CA_NTD_ from the crystal structure of the CypA-CA_NTD_ complex (PDB: 1AK4) (ref. 11) with the CA_NTD_ in our rigid body fitted model (3J4F) places the CypA model exactly into the CypA density region (Fig. 2e). Most interestingly, the CypA density appears to bridge two neighbouring CA hexamers and is located directly above the CA_CTD_ dimer interface (Fig. 2e) of two adjacent, non-CypA-binding CA molecules (Fig. 2c–e; Fig. 3e, red CA molecules).

An initial interpretation may be that the bridging density corresponds to a CypA dimer, with each CypA molecule binding to the CypA-binding loop from the adjacent CA hexamer. However, there is not enough space to accommodate two CypA molecules without extensive clashing. In addition, analytical centrifugation experiments suggest that CypA is primarily monomeric in our experimental conditions (Supplementary Fig. 3). Furthermore, focused classification using reference-based cross-correlation to separate CypA-binding modes into distinct classes, resulted in class averages that contain monomeric CypA bound to CA hexamers (Supplementary Fig. 2c). Thus, the bridging density likely corresponds to a single-CypA molecule, in which case, one may ask why CypA only occupies one particular helical array, rather than being equally distributed along all directions. One possibility is that a single CypA interacts with two CA molecules that are arranged in a specific geometry, with each CA molecule recognizing a different region of the enzyme. Supporting evidence for this idea comes from two sources: (1) a crystal structure of the CypA/CsA complex (PDB: 2RMA), in which the opposite faces of CypA each bind a molecule of CsA (ref. 31); and (2) an analysis of the inhibition of CypA-Gag interaction by CsA, showing a sigmoidal CsA inhibition curve, indicating synergy between two CsA in the inhibition^12^. For these reasons, we suggest that a single-CypA molecule spans two CA hexamers in the tubular complex (Fig. 2e).

### Computational modelling of the CypA-CA lattice

For the binding of a single-CypA molecule to two adjacent CA hexamers in a helical array, two binding modes are possible: (1) CypA binds more strongly to the left hexamer, extending across to the right one; or (2) CypA binds the other way around. The density map is an ensemble average of all possible binding modes from many tubes and, thus, cannot distinguish between the binding modes. Therefore, we carried out MD flexible fitting (MDFF) to model the interaction between CypA and the CA tubes (simulations 1and 2—Supplementary Table 1), monitoring, during the simulation, the cross-correlation between the model and the experimental density (Supplementary Fig. 4). An ensemble of 10 fully saturated CypA-CA tubular complex models (Fig. 3a) was constructed as described in Supplementary Information. Overlapping or clashing CypA molecules were removed with no bias from the saturated models, resulting in 10 tubular complex models with thousands of statistically independent arrangements of CypA over the CA lattice (Supplementary Fig. 5a). Interestingly, in all cases, clashes between CypA and adjacent CA molecules were only observed along the least curved helical direction of the tube (a typical HIV tube contains a CA lattice with three helical directions along which repeating arrays are found, each direction with different characteristic curvatures as illustrated in Fig. 2c and Supplementary Fig. 6), resulting in removal of CypA molecules along this direction (Supplementary Fig. 5a). Subsequently, the CypA-CA tubular complex models were equilibrated for 100 ns (Supplementary Fig. 7, simulation 2—Supplementary Table 1), and simulated electron density maps were generated for each model (Supplementary Fig. 5b). Mimicking the cryoEM real-space helical reconstruction process, a final, averaged density map with helical symmetry imposed was calculated from all these models.

The resulting average density map (Fig. 3b) matches the experimental map remarkably well, especially the selectivity of CypA for the most curved CA lattice array, with a cross-correlation value of 0.967 (Figs 2c and 3b). During the course of the MD simulations (simulation 2), the CA lattices remained rigid, as previously reported^30^, whereas the CypA-binding loops and the CypA molecules exhibited a range of motions (as calculated from root-mean-square fluctuations of the atomic positions, mapped onto the structure of the tube in Supplementary Fig. 8), which can be considered a likely cause for the lower resolution of the cryoEM density map at the CypA region (Supplementary Fig. 1b,c). Indeed, principal component analysis of longer trajectories of CypA-CA subsystems extracted from the MDFF-derived tube (simulations 4 and 6, Supplementary Fig. 9), revealed two distinct types of motion depending on whether CypA is bridging along the most curved CA array or not (Fig. 3c,d). Although the bridging CypA exhibits limited motion (1°) perpendicular to the array direction and appears locked onto the curved CA lattice (Fig. 3c), disjointed CypA is more mobile (20°) and inter-converts between the two conformers observed in the crystal structure of the CypA-CA_NTD_ complex (Fig. 3d, Supplementary Movie 1)^11^.

To investigate the detailed interactions between CypA and CA, a subsystem comprising two adjacent CA subunits with a corresponding bridging CypA (Fig. 3e, Supplementary Table 1 and Supplementary Movie 2). The MD simulation revealed, beside the first binding interface at the canonical CypA-binding site, a second binding interface between CypA and CA, comprising residues A88, G89 and P90 from CA, and A25, P29 and K30 from CypA (Fig. 3f, site 2). The interaction at the second, non-canonical CypA-binding site is weaker than at the canonical binding site, as shown from pair interaction energies −16.1±7.3 and −28.9±17.0 kcal mol^−1^, respectively, obtained from unrestrained CypA simulations (simulation 4—Supplementary Table 1). However, the second site, properly positioned, could yield a strong binding avidity once CypA binds to the first CA through the canonical site. Interestingly, a similar second binding site, involving P29 and K30 on CypA, was observed in an early crystal structure of the CypA/CsA complex (PDB: 2RMA), in which two CsA molecules bind CypA (Fig. 3g)^31^. We conclude, therefore, that CypA likely employs both binding sites to strengthen the CA dimer interface in the CA assembly, thereby stabilizing the viral capsid.

### Solid-state NMR spectroscopy

**Figure 4:**
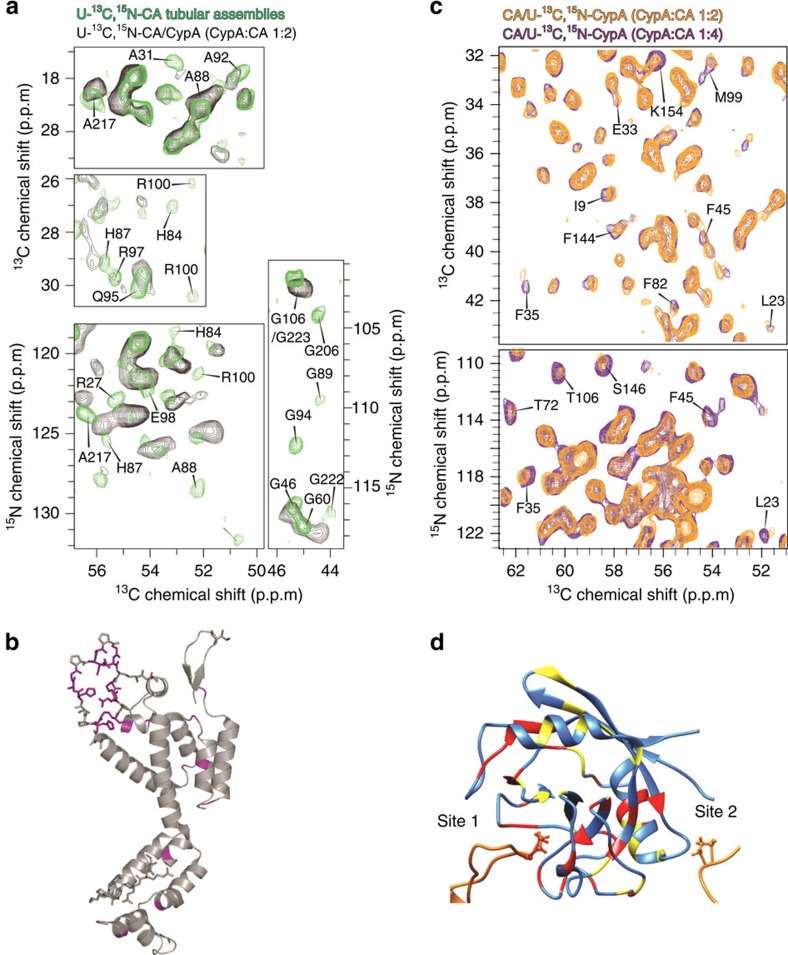
Solid-state MAS NMR studies of the intermolecular interfaces formed between CypA and CA in CypA-CA complex assemblies. (**a**) Expansions of 2D MAS NMR spectra of U-^13^C,^15^N-CA tubular assemblies, free (green) and in complex with CypA (black), acquired at 19.9 T. Top two panels: ^13^C-^13^C CORD, bottom two panels: ^15^N-^13^C NCACX. The peaks whose chemical shifts or intensities change in the presence of CypA are labelled. (**b**) Mapping of chemical shift and/or intensity changes onto the 3D structure of CA (PDB: 3NTE). Purple indicates residues whose shifts or intensities are perturbed. (**c**) Expansions of 2D MAS NMR spectra of CA/U-^13^C,^15^N-CypA assemblies acquired at 19.9 T. Top: ^13^C-^13^C CORD, bottom: ^15^N-^13^C NCA. The spectra are shown for the varying CypA:CA ratios: 1:4 (purple), and 1:2 (orange). The peaks whose chemical shifts or intensities change as a function of the CypA:CA ratios are labelled. (**d**) Mapping of chemical shift and/or intensity changes on the 3D structure of CypA. Red and yellow are the changes when the CypA:CA ratio is shifted from 1:4 to 1:2 and 1:2 to 1:1, respectively.

To experimentally delineate the CA and CypA residues that form the intermolecular CypA-CA interface, we employed magic angle spinning (MAS) NMR spectroscopy. We previously showed that CA tubular assemblies yield unprecedented high-resolution MAS NMR spectra, permitting their in-depth characterization^32^. In complex with CypA, multiple chemical shift perturbations were detected in the two-dimensional (2D) COmbined R2-Symmetry Driven and NCACX spectra from isotopically labelled CA (U-^13^C,^15^N) tubular assemblies (Fig. 4a, Supplementary Fig. 10). The perturbed resonances belong mostly to residues in the CypA-binding loop (Fig. 4b), in agreement with the cryoEM results. Additional perturbations, observed for a few residues located in other CA regions (Fig. 4b), are likely due to allosteric effects. Our recent study revealed that binding of CypA to tubular assemblies of CA results in significantly attenuated mobility of CypA loop and other CA regions^33^.

To infer the CypA residues that interact with the assembled CA, we prepared isotopically labelled CypA (U-^13^C,^15^N) with assembled CA tubes at three different CypA:CA input ratios of 1:4, 1:2 and 1:1, and recorded CORD and NCA spectra (Fig. 4c, Supplementary Fig. 10). Numerous chemical shift perturbations and/or intensity changes were observed as the CypA:CA ratio increased (Fig. 4c, Supplementary Table 2). Many of the spectral changes correspond to the CypA residues comprising the canonical binding site. In addition, we observed many residues, not associated with the canonical binding site, that experience spectral changes. Mapping these residues onto the CypA structure reveals that many are located at or in the vicinity of the non-canonical second binding site (Fig. 4d), consistent with the results of cryoEM and MD simulations. Interestingly, at a CypA:CA ratio of 6:6, additional spectral perturbations are found in residues distal to either the canonical or the non-canonical binding site (Fig. 4d). These perturbations are likely due to allosteric effects at high CypA concentrations rather than direct interactions with CA. We note that the NMR experiments were run for co-assembled samples (Fig. 1a–c). The observed enhanced binding (not saturated at sub-stoichiometric conditions) in these highly curved assemblies (as opposed to the tubular assemblies) is consistent with our model that one CypA binds the CA hexamer along the curved direction of CA tubes. The MAS NMR results corroborate the cryoEM findings and computer simulations, revealing that CypA binds to assembled CA through canonical and non-canonical binding sites.

## Discussion

A significant result from this study is the discovery of a non-canonical, second site of CypA for binding to the assembled CA lattice. Through this site, CypA bridges two CA molecules from adjacent hexamers, thus stabilizing the CA lattice (Fig. 1a). The additional interaction interface that is established by the new site further strengthens the binding of CypA to the CA lattice through avidity. CypA is known to have different effects on viral infectivity in different host cells; some are enhancive while others are restrictive^24^. Whether these effects correlate with CypA's cellular expression level or are associated with an unknown cell-specific cellular factor is still not clear. The fact that CypA, via its second binding site, binds selectively along the most curved CA array (Fig. 2c) and promotes curved assembly (Fig. 1c), further implies that CypA and proteins containing CypA domains are best suited to interact with a conical capsid exhibiting a continuous curvature.

We found that sub-stoichiometric amounts of CypA (up to 3:6 CypA:CA) stabilize the CA assembly (Fig. 1d). On the other hand, a high level of CypA reduces capsid stability (Fig. 1d) and disrupts the capsid assembly (Fig. 1a)^28^. These seemingly contradictory observations can be explained by steric hindrance between two CypA molecules upon simultaneous binding to neighbouring CA hexamers, thus weakening, at high concentration, the dimer assembly interface. This effect is utilized by the host restriction factors Trim-Cyp and Trim5α to carry out their function^34^,^35^,^36^; both proteins contain stable dimeric capsid-binding motifs, CC-Cyp and CC-SPRY, in addition to the B-box domain for higher-order oligomerization, to enhance the affinity of interaction through avidity effects^37^. The CC-SPRY dimer, not the SPRY monomer, effectively binds to and breaks the capsid between CA hexamers^38^,^39^. Our data further suggest a plausible mechanism for Trim-mediated premature uncoating and viral restriction that involves direct binding of Trim-Cyp and Trim5α at the inter-hexamer interface and subsequent destabilization of the viral capsid^37^,^40^,^41^. Since CypA prefers curvature and stabilizes curved assemblies, the variable curvature in the mature core would help CypA binding. CypA binding may also have a greater effect on a continuously curved capsid. CypA binding to the HIV-1 capsid also enhances the antiviral activity of some small molecule inhibitors, such as PF74 (ref. 42). Based on our observation that CypA binding hardens the capsid lattice, in our AFM experiments, we suggest that PF74, which appears to act at the uncoating step of infection, acts more effectively on CypA-stabilized capsid.

**Figure 5:**
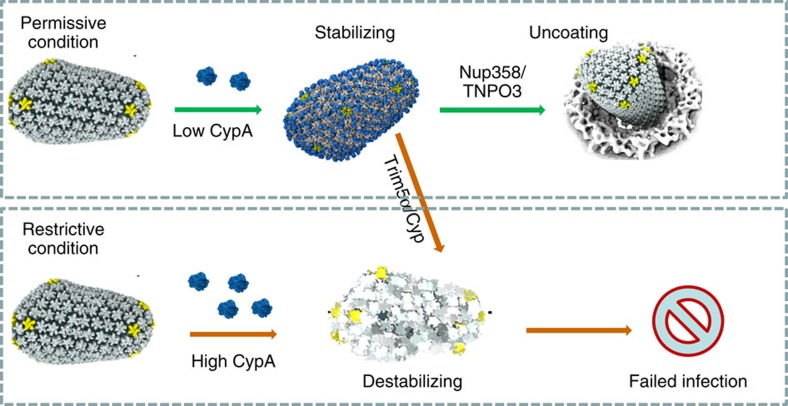
Model for CypA's role in HIV-1 infection. In permissive cells, sub-stoichiometric levels of CypA stabilize and protect the viral capsid moving in the cytoplasm as it moves towards the nuclear pore, where Nup358 additionally binds to the capsid and promotes uncoating. In non-permissive cells, either high levels of CypA or CypA in combination with host restriction factors such as Trim5α and Trim-Cyp facilitate premature uncoating and result in viral restriction.

What are the functional consequences of CypA binding to the HIV-1 core? Lentiviruses, such as HIV-1, are differentiated from other retroviruses by having evolved the ability to traverse the nuclear pore complex, allowing replication in non-dividing cells. HIV-1 has been suggested to use CypA and CPSF6 to cloak its genome during migration through the cytoplasm, allowing evasion of innate immune sensing in primary human macrophages and dendritic cells^4^,^5^,^43^,^44^. Our data support this notion and suggest that the second interaction interface is important for the protective role of CypA to the viral capsid. On the basis of previous studies by others^8^ and our current results, we propose a simplified model for the function of CypA in HIV-1 infection (Fig. 5). In permissive cells, CypA affects HIV-1 transduction in two stages. The first stage is within the cytoplasm during reverse transcription. Since a single CypA bridges two CA molecules from adjacent hexamers, a sub-stoichiometric level of CypA stabilizes the capsid and protects the viral core from premature uncoating, thus promoting reverse transcription in all cell types^34^. At the second stage, once the HIV-1 core reaches the nuclear membrane, the CA subunits that are not bound by CypA could be available for binding to Nup358 (ref. 24), thus facilitating HIV-1 infection by coordinating proper uncoating of the core in target cells^6^,^45^. On the other hand, a high level of CypA binding or addition of Trim-Cyp/Trim5α could destabilize the capsid^18^,^46^,^47^; CypA enhances Trim-mediated restriction at a stage after reverse transcription in restrictive cells^48^, and productive restriction can occur even when a fraction of CA in the capsid is available for Trim-Cyp binding^49^. Taken together, our results provide new insights into how CypA engages in modulating HIV-1 infectivity.

In summary, we used an integrative multi-disciplinary approach, combining cryoEM, computer modelling and MD simulations, solid-state NMR and biochemical analysis, to investigate the interactions between the host cell factor CypA and HIV-1 capsid assembly. We uncovered a novel interaction interface of CypA which may provide a new avenue for the development of therapeutic interventions that target CypA interactions with the HIV-1 capsid.

## Methods

### Protein expression and purification (CA-FL and CypA)

Recombinant wt HIV-1 CA (HXB2) and CypA proteins were expressed in *Escherichia coli*, Rosetta 2 (DE3), cultured in Luria-Bertani media or modified minimal medium, and induced with 0.4 mM IPTG at 23 °C for 16 h (refs 50,51). Natural abundance and U-^13^C, ^15^N isotopically labelled CA proteins were purified over a 5 ml Hi-Trap QP column (GE Healthcare, Piscataway, NJ, USA) in 25 mM sodium phosphate (pH 7.0), 1 mM DTT and 0.02% sodium azide, followed by a 5 ml Hi-Trap SP columns (GE Healthcare) using a 0.1 M NaCl gradient in a 25 mM sodium phosphate (pH 5.8), 1 mM DTT and 0.02% sodium azide mixture. The final purification step comprised gel-filtration over Hi-Load Superdex 200 16/60 columns (GE Healthcare) in 25 mM sodium phosphate (pH 6.5), 100 mM NaCl, 1 mM DTT and 0.02% sodium azide. Proteins were concentrated to 10 mg ml^−1^ using Amicon concentrators (Millipore, Billerica, MA, USA), flash-frozen with liquid N2, and stored at −80 °C. Cells expressing unlabelled and U-^13^C, ^15^N isotopically labelled CypA were lysed using sonication in 25 mM sodium phosphate buffer at pH 7.0, and centrifuged at 15,000 r.p.m. at 4 °C for 1 h. After adjusting the pH to 5.5 with acetic acid, the solution was centrifuged again at 15,000 r.p.m. at 4 °C for 1 h, and the supernatant was loaded onto a 5 ml HiTrap SP HP (GE Healthcare) column and eluted with a 0–1 M NaCl gradient. Fractions containing the target protein were combined and further purified with a Hi-Load Superdex 75 pg 26/600 (GE Healthcare) column in a buffer containing 25 mM sodium phosphate, 1 mM DTT and 0.02% NaN_3_, pH 5.5.

### CA assembly and CypA-binding assays

For CA and CypA co-assembly, 80 μM (2 mg ml^−1^) wt CA was mixed with CypA at different molar ratios in a buffer containing 2.25 M NaCl and 50 mM Tris-Hcl (pH 8.0) and incubated at 37 °C for 1 h. For CypA binding to pre-assembled CA tubes, 10 mg ml^−1^ wt CA was first diluted to 2 mg ml^−1^ in a buffer containing 1 M NaCl, 50 mM Tris pH 8.0 and incubated at 37 °C for 1 h. The assembly mixture was then incubated with CypA at different molar ratios at room temperature for 2 h. At the end of incubation, 5 μl samples were immediately used for cryoEM analysis. An amount of 6 μl samples from the same reaction mixtures were mixed with 4 × LDS loading buffer (Invitrogen, NP-0007) supplemented with 10 mM DTT for SDS–polyacrylamide gel electrophoresis (SDS–PAGE) analysis (t). The remaining sample was pelleted at 20,000*g* with an Eppendorf centrifuge 5417R for 15 min, and supernatants (s) and resuspended pellets (p) were mixed with 4 × LDS loading buffer for gel analysis. Total, supernatant and pellet samples, without boiling, were fractionated on 10% SDS–PAGE and stained with Coomassie Blue. Each experiment was carried out at least three times.

To determine the binding ratio of CypA:CA, the SDS–PAGE gels were scanned using an Epson 4990 scanner. The integrated intensities of CA and CypA protein bands were measured using Image J 1.40 program (NIH). The molar ratios were calculated according to the formula (CypA intensity/CypA molecular weight)/(CA intensity/CA molecular weight) and calibrated using the input ratios as standards.

### AFM indentation experiments

For AFM measurements, 160 μM wt CA was dialysed against a high-salt buffer (50 mM Tris-Hcl pH 8.0, 1 M NaCl) overnight at 4 °C. The assembled protein was diluted to 80 μM and incubated with varying concentrations of CypA (13, 40 and 80 μM) for 2 h at 4 °C. CA assemblies were attached to hexamethyldisilazane-coated microscope glass slides using previously described methods^52^,^53^. All measurements were conducted in a fluid environment (high-salt buffer). The experimental system consisted of a Nanowizard ULTRA Speed atomic force microscope (JPK, Berlin, Germany) mounted on an inverted optical microscope (AxioObserver, Carl Zeiss, Heidelberg, Germany). Images of capsid particles were acquired in AFM tapping mode. Silicon nitride probes (DNP, *k*=0.1±0.01 N m^−1^, Bruker) were used, their spring constants being determined experimentally by measuring the thermal fluctuations of the cantilevers (Hutter and Bechhoefer, 1993). To determine the stiffness of an individual capsid, an indentation experiment was performed. For each capsid, ∼100 successive force-distance curves were collected at a scan rate of 20 Hz. To maintain a constant range of indentation depths, the maximal loading force was limited to a range of 0.2–1.5 nN, yielding maximal indentation depths of 4 nm. Capsid stiffness was calculated from the slope of the force-distance curve, according to Hooke's law, on the assumption that the capsid and the cantilever can be modelled as two springs arranged in series.

### CryoEM specimen preparation and data collection

Full-length wt CA (10 mg ml^−1^) was diluted to 2 mg ml^−1^ in high-salt buffer (1 M NaCl, 50 mM Tris pH 8.0) at 37 °C for 1 h for tubular assembly. The assembly mixture was then incubated with CypA (27 μM) at room temperature for 2 h. CryoEM grids were prepared according to the procedure described previously^30^. Low-dose (∼15 e^−^ Å^−2^) projection images were collected with a Tecnai TF20 microscope (FEI Corp., OR) operated at 200 kV, on Kodak SO163 films at a nominal magnification of 55,000, with under-focus values ranging from 1.0 to 3.5 μm. The best micrographs were selected and digitized using a Nikon super coolscan 9000 ED scanner (Nikon, Japan) at an optical resolution of 4,000 d.p.i.

### 3D EM reconstruction

More than 800 films of tube images possessing a wide range of diameters and helical symmetries were collected. Nineteen tubes with (−8, 13) symmetry were selected and boxed into segments of size 512 × 512 pixels with a shift of two subunits along the helical axis by the EMAN BOXER program (ref. 54). The defocus value of each micrograph was evaluated using the program CTFFIND3 (ref. 55). An initial density map was reconstructed from binned segments of 256 × 256 pixels using iterative helical real-space reconstruction^29^. The refined helical parameters corresponded to a rise of 7.393 Å and a rotation angle of 138.13°. The preliminary map was further refined using iterative helical real-space reconstruction without binning. During the refinement, helical symmetry and full-contrast transfer function (CTF) correction were applied. A total of 8,640 helical segments, containing 17,280 asymmetric units, were included in the final reconstruction. The Fourier shell correlation curve was calculated using gold standard procedure from two independent half data sets.

### Focused classification

To accurately assess a given CypA-CA binding site for CypA occupancy, reference-based classification was used. Owing to the very large mass discrepancy between the rest of the CypA-CA tube and an individual CypA monomer or dimer, very accurate background subtraction was needed, essentially as described in ref. 56.

References were generated by simulating electron density maps for CypA from the CypA-CA_NTD_ crystal structure (PDB: 1AK4) filtered to 12 Å using Chimera^57^. These were then aligned to the final EM map in three binding modes: CypA monomer binding to the left hexamer; CypA monomer binding to the right hexamer; CypA composite dimer (referred to as CypA dimer hereon) binding to both, with steric clashes present. In addition, we included a negative control with a CypA dimer positioned above the centre of the CA hexamer in question; any significant density showing up in the results would indicate a bias selecting for noise. The cross-correlation between each of these four references was calculated for each particle, in replicates of three as described in the following.

To account for the large difference in signal-to-noise ratio between the re-projections from the final model and its individual data projections, we added noise and information from the particle's CTF for each comparison. Three hundred noise images were selected from different micrographs near the tube density to provide the most accurate combination of structural and detector related noise. Three noise images were selected at random for each comparison, convolved with the particles CTF, centred to a mean of zero, and normalized by the subsequent images intensity range. To each of these images, a re-projection of the final EM map at the given particles orientation was added (also convolved with the particles CTF), centred and normalized. These three replicates were individually subtracted from the data projection, everything being low-pass filtered to 12 Å. Finally, the three difference images were masked off using a binary mask derived from the projection of the corresponding CypA dimer reference (one for the dimer and both monomer-binding modes, and one for the negative control).

The average CCC from the three replicates was calculated for all four binding modes and used as the metric for classification. Those with a CCC > 0 with the dimer reference were initially separated from the full data set. This subset was then further split in two, based on the difference between the left (‘up') and right (‘down') binding modes, with a CCC_up_ - CCC_down_ > 0 indicating an ‘up' class. No particles were excluded from the analysis and no further alignment was introduced. The classified maps were directly derived from the pre-aligned tube segments that have been sorted into different classes.

### Molecular dynamics flexible fitting

Ten tubular CypA-CA complexes were derived as described in Supporting Information, and further refined using MDFF, as follows. Ions near the surface of the protein were placed using cionize^58^. Bulk water and Na/Cl ions were then added using visual molecular dynamics (VMD)^58^, setting the total concentration of NaCl to 1 M. Each of the resulting systems contained 1,900,000 protein atoms and ∼25,600,000 total atoms, including solvent. The systems were equilibrated at 310 K and 1 atm for 5 ns while applying positional restraints on all heavy atoms of the protein. MDFF^59^ was then applied for 10 ns with a grid scaling of *ϕ*=0.1 coupled only to backbone heavy atoms. Domain restraints were applied to maintain the structural integrity of each individual CypA-CA_NTD_ complex. Additional restraints were applied in the form of extra bonds to maintain secondary structure integrity and to prevent transitions of *cis*/*trans* bonds and chirality errors^59^. MDFF was performed using NAMD 2.9 (ref. 60) with the CHARMM22-CMAP force field^61^,^62^. Simulated density maps needed for determining cross-correlation between model and EM data, were generated using VMD^58^.

### Molecular dynamics simulations of CypA-CA complexes

All-atom MD simulations of reduced systems for the two types of CypA-CA complexes, namely bridged and disjoined, were performed. For this purpose, ions in close proximity to the protein were placed using cionize. Bulk water and Na/Cl ions were then added using VMD, setting the total concentration of NaCl to 1 M. The systems were then equilibrated at 310 K and 1 atm for 10 ns using the NAMD 2.10 and CHARMM36FF (ref. 63) together with the TIP3P water model^64^, corresponding to simulations 3 and 5 in Supplementary Table 1. Simulations of CypA-CA complexes were then performed, again employing NAMD 2.10, but with the polarizable CHARMM force field based on the classical Drude oscillator model^65^,^66^ together with the SWM4-NDP water model^67^ at 310 K and 1 atm (simulations 4 and 6 in Supplementary Table 1).

All simulations carried out in the present study used the r-RESPA integrator available in NAMD. Long-range electrostatic force calculations employed the PME method (particle-mesh Ewald), utilizing a grid spacing of 2.1 Å and eighth order interpolation, with a 1.2 nm cutoff. Simulations using the CHARMM additive force field, employed a time-step of 2 fs, with non-bonded interactions evaluated every 2 fs and electrostatics updated every 4 fs; all hydrogen bonds were constrained with the SHAKE algorithm. For the simulations employing the CHARMM Drude polarizable force field (simulations 4 and 6), the systems were first equilibrated using CHARMM36 for 10 ns at 310 K and 1 atm (simulations 3 and 5), then the Drude particles were added using ‘drude prepper' as implemented in CHARMM-GUI^68^. All systems were minimized for 20 steps, and then equilibrated at 310 K and 1 atm for 1 ns while maintaining positional restraints for the proteins' heavy atoms with an integration time step of 0.5 fs. Screened Coulomb corrections of Thole were calculated within a cutoff of 5 Å. Restraints were applied to a Drude oscillator if its length exceeded 0.2 Å, assuming a force constant of 40000, kcal Å^−2^. Production runs were performed with a time step of 1 fs, with non-bonded interactions evaluated every 1 fs and electrostatics updated every 1 fs. Temperature of the Drude particles was maintained at 1 K.

### Computational modelling of CypA-CA tubular complexes

A helical tube of CA was constructed by docking the MDFF-derived hexameric structure (PDB: 3J34), using Chimera, to each of the hexamers in the experimental tubular density with (−8, 13) helical symmetry. Reverse mutations, L6I, L83V, E92A and H120N, were applied to match the wt (HXB2) sequence. Protonation states of titratable groups were calculated using PDB2PQR (ref. 69). The tube was decorated with CypA employing VMD^70^, as described below. Two binding modes of CypA to CA are observed in the CypA-CA_NTD_ crystal complex and, accordingly, in building the tube model for each of the CA monomers in the lattice, one of the two CypA conformers was selected, at random with equal probability. The location of the CypA molecule was derived by aligning the CA_NTD_ domain of each CA in the tubular lattice with the CA_NTD_ of the CypA-CA_NTD_ crystal structure (PDB: 1AK4) (ref. 11) using root-mean-square fitting, with excellent agreement (C_α_-RMSD of 0.5 Å). Coordinates were taken from the selected conformer, including the coordinates of the CA loop binding to CypA. The procedure was repeated 10 times, to explore the combinatorial complexity of the binding of CypA to CA. The resulting 10 fully saturated CypA-CA tubes contain extensive clashes between neighbouring CypAs. To correct for steric clashes, CypAs were removed from each of the saturated models, following a random walk on a self-avoidance path over the lattice. At each step of the removal procedure, a CypA was chosen at random, and, if clashes between the selected CypA and adjacent CypAs were present, then the adjacent CypA molecule(s) was (were) removed from the model. The procedure was repeated over the set of remaining CypAs—those that had not been removed or selected on a previous step—yielding a model with no clashes.

### Solid-state NMR specimen preparation

Natural abundance CA and U-^13^C,^15^N-labelled CypA proteins were concentrated to 30 mg ml^−1^ in 25 mM phosphate buffer (pH 5.5), and then mixed at varying CypA:CA ratios (1:4, 3:6 and 6:6). Assemblies of CypA-CA were prepared by adding NaCl to a final salt concentration of 2.4 M, followed by one-hour incubation at 37 °C. Assembled CypA-CA were pelleted by centrifugation at 50,000 r.p.m. at 4 °C and packed into three 3.2 mm thin-wall Bruker rotors. The amount of U-^13^C,^15^N-labelled CypA in each rotor was ∼0.15 μmol.

### MAS NMR spectroscopy

2D MAS NMR spectra of CypA-CA assemblies were acquired on a Bruker 19.9 T spectrometer with a 3.2 mm HCN Efree probe. Larmor frequencies were 850.400 MHz for ^1^H, 213.833 MHz for ^13^C, and 86.170 MHz for ^15^N. 2D CORD and NCA spectra were collected at the MAS frequency of 14.000±0.001 kHz, regulated by a Bruker MAS controller, and the temperature was maintained at 4.0±0.5 °C throughout the experiments by a Bruker temperature controller.

Typical pulse lengths were 2.6 μs (^1^H), 3.4 μs (^13^C) and 3.7 μs (^15^N); ^1^H-^13^C cross-polarization was performed using a linear amplitude ramp, with the ^1^H rf field of 88 kHz matched to the second spinning sideband. The ^1^H-^13^C and ^1^H-^15^N contact times were 0.6 and 1.1 ms, respectively. The ^15^N-^13^C DCP contact time was 6 ms. Small phase incremental alternation ^1^H decoupling (95 kHz) was applied during the evolution and acquisition periods. The modulated ^1^H field strengths during CORD were 14 and 7 kHz, and the CORD mixing time was 50 ms. Under these conditions predominantly one-bond correlations are observed in the spectra. Time-proportional phase incrementation was used for frequency discrimination in the indirect dimension.

### Processing and analysis of solid-state NMR spectra

All spectra were processed with NMRpipe and analysed using Sparky (https://www.cgl.ucsf.edu/home/sparky/). 30°-shifted, for CORD spectra, or 45°-shifted, for NC spectra, sine bell apodization followed by a Lorentzian-to-Gaussian transformation was applied in both dimensions. Forward linear prediction to twice the number of the original data points was used in the indirect dimension, followed by zero filling to twice the total number of points.

### Analytical centrifugation

Sedimentation velocity experiments were performed in an XL-A analytical ultracentrifuge (Beckman Coulter) in two-channel Epon centrepieces. The data were collected at 280 nm and a speed of 40,000 r.p.m. in an An60Ti four-hole rotor at 25 °C. The concentration of protein loaded was approximately 0.35 mg ml^−1^. The data were analysed with the program SEDFIT using the c(s) and c(s,ff0) models (http://www.analyticalultracentrifugation.com/default.htm). The c(s) model was used for molecular weight estimation of the sedimenting species. The buffer density, buffer viscosity, and protein partial specific volume used in these calculations were estimated with the program SEDNTERP (http://bitcwiki.sr.unh.edu/index.php/Main_Page).

## Additional information

**Accession codes:** CryoEM structural data have been deposited at the EMDB under accession code EMD-3075 and EMD-3076, and the MD atomic model of the CypA-CA complex has been deposited at the Protein Data Bank under accession code 5FJB.

**How to cite this article:** Liu, C. *et al*. Cyclophilin A stabilizes the HIV-1 capsid through a novel non-canonical binding site. *Nat. Commun.* 7:10714 doi: 10.1038/ncomms10714 (2016).

## Supplementary Material

Supplementary Information Supplementary Figures 1-10 and Supplementary Tables 1-2

Supplementary Movie 1 All-atom MD simulation of the CypA/CA complex, which comprises one CA molecule (orange) and one CypA molecule (blue) within a tubular configuration. The movie illustrates the interconvertion between the two CypA conformers observed during the simulation. Shown in the inset is the time evolution of the root-mean-square deviation of the simulation from each of the conformers, red and blue respectively, in the crystal (1AK4).

Supplementary Movie 2 All-atom MD simulation of the CypA-CA complex comprising two CA molecules (orange) and one CypA (blue) within a tubular configuration. The movie illustrates the two CA binding sites on CypA: site 1 is the canonical binding site and site 2 is the non-canonical binding site. Selective residues at the site 2 interface are labeled.
