# Supplementary material for: Cyclophilin A stabilizes the HIV-1 capsid through a novel non-canonical binding site
Source: Nat Commun. 2016 Mar 4;7:10714. doi: 10.1038/ncomms10714 (PMC4785225; doi:10.1038/ncomms10714)
Supplement: Supplementary Information — Supplementary Figures 1-10 and Supplementary Tables 1-2 [file ncomms10714-s1.pdf]

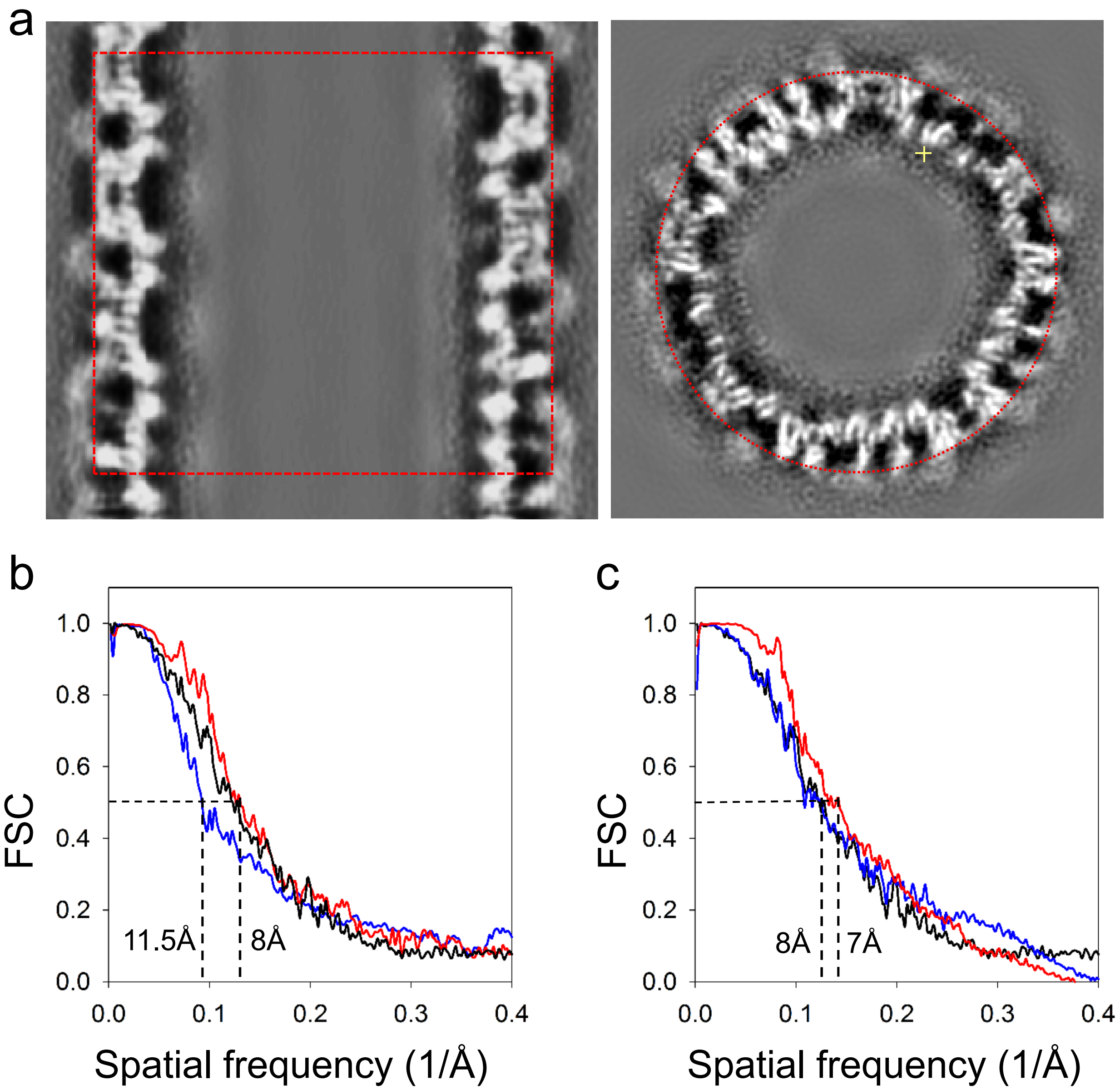

Figure S1. Iterative real space helical reconstruction of CypA-CA tubular assemblies. (a) Cross-sectional (left) and axial (right) views of the CypA-CA assembly density map. The sub-region corresponding to CA is indicated with a red box and circle. (b) Comparison of gold-standard FSC curves of the CypA-CA density map calculated from the entire tube (black), the CA region (red) and the CypA region (blue). (c) Comparison of gold-standard FSC curves of the reconstructions with particle alignment based on the whole tube (black), CA region (red), and the CypA region (blue).

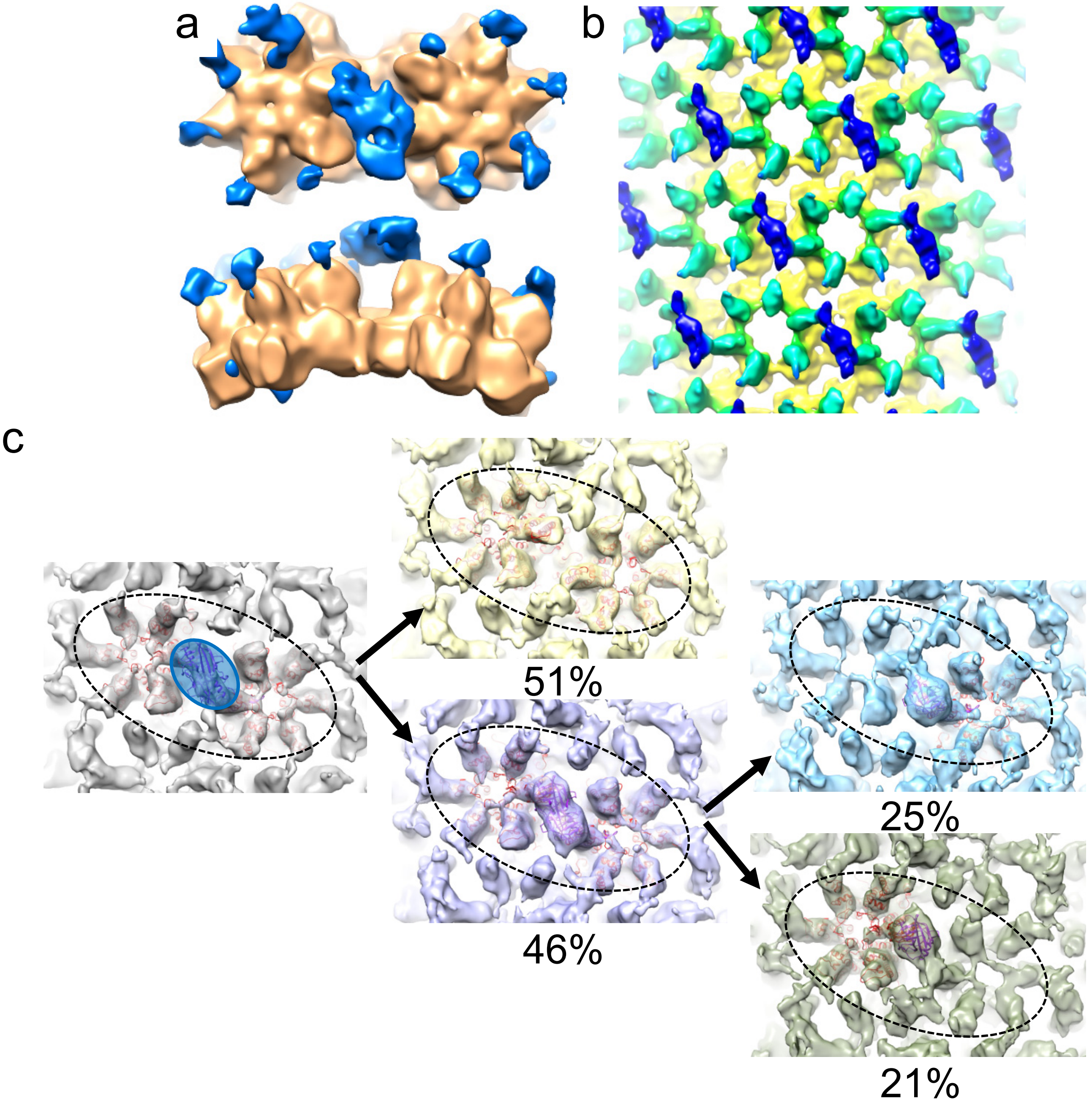

Figure S2. (a) A difference map (blue) calculated for CypA-bound and free CA tubes, superimposed onto the unliganded CA map (gold). Since the CypA-bound and CypA-free maps are from different helical families, two CA hexamers were cut out for the difference map calculation. Top and side views are shown. (b) Density map of CypA-CA complexes of a second helical family (-12,11). (c) Reference-based focused classification of the CypA binding modes. Left, the final averaged map (as shown in Fig. 2c) contoured at 1.5σ. Dashed-black oval indicates two CA hexamers with a bridging CypA density (blue, focus area). Middle, two class averages without CypA density (top) and with CypA density (bottom). The percentages of contributing images are indicated below. Right, two class averages with CypA bound to the right CA hexamer (top) and to the left CA hexamer, respectively. CA hexamer model (PDB code 3J34) is docked into the density map and CypA-CA-NTD complex model (PDB code 1AK4) was placed by aligning its CA-NTD to the docked 3J34.

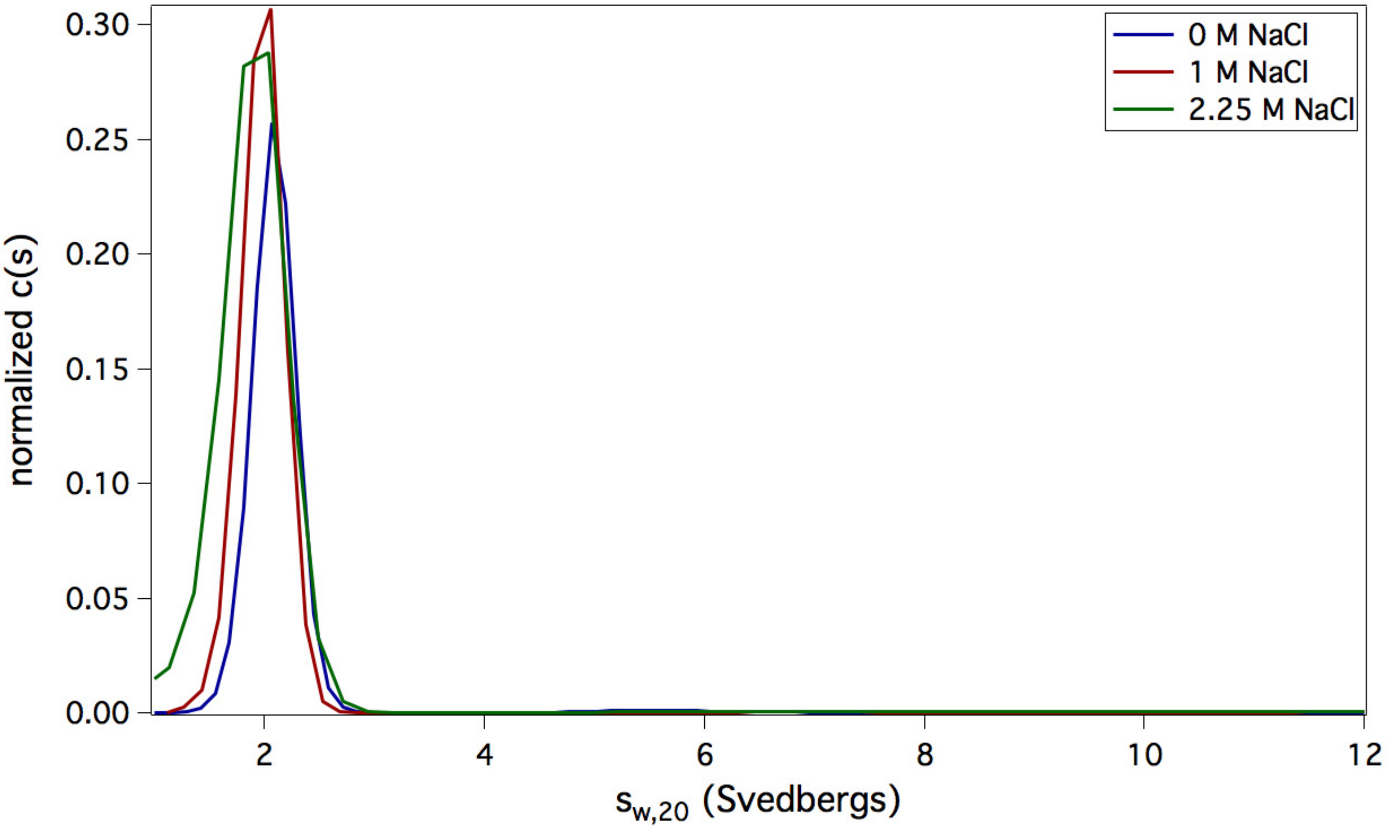

Figure S3. Sedimentation coefficient distribution (c(s) vs $s_{20,w}$) of cyclophilin A as a function of ionic strength. CypA at a loading concentration of approximately 0.35 mg/ml was supplemented with 0 M (blue), 1 M (red), and 2.25 M (green) NaCl. Samples were centrifuged at 40,000 rpm and 25 °C in a Beckman XL-A analytical ultracentrifuge. Absorbance data were acquired at 280 nm. c(s) profiles were calculated using the program SEDFIT. The buffer density, viscosity, and protein partial specific volume were estimated using the program SEDNTERP. Molecular weight values of 18.3, 17.0, and 17.1 kDa for the 0 M, 1 M, and 2.25 M NaCl samples, respectively, correspond well with the theoretical molecular weight of 17.8 kDa for the CypA monomer.

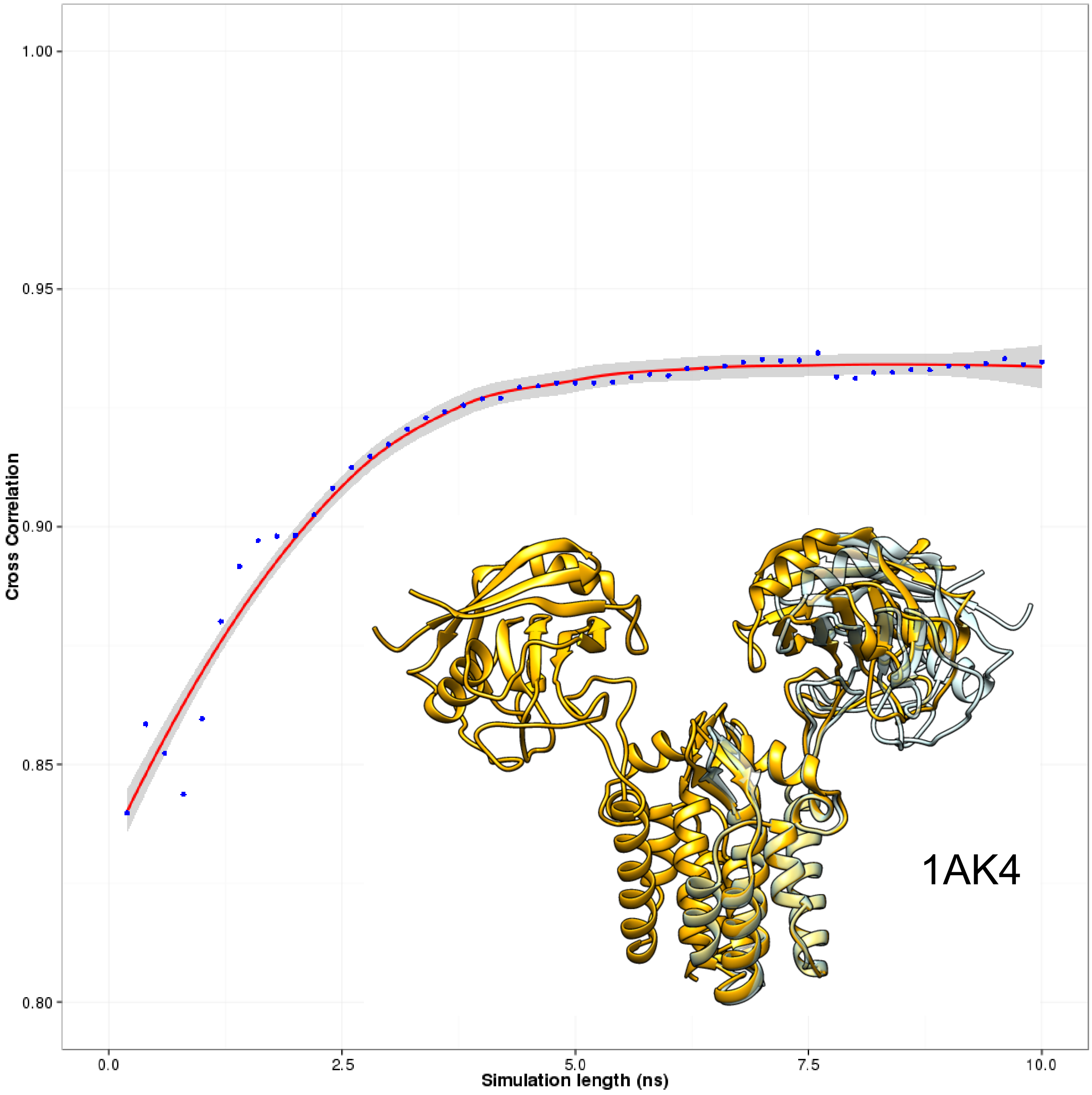

Figure S4. Cross-correlation between all-atom model and experimental density (blue dots) as the MDFF simulation progresses (Table S1 - Simulation 1). The simulation reaches a plateau after 5.0 ns. The red line shows the cross-correlation fitted to a generalized additive model, and highlights the convergence of the MDFF simulation. Inset, two binding conformations of CypA to CA as observed in the crystal structure 1AK4 (gold), with an overlay of these two (gold and transparent blue).

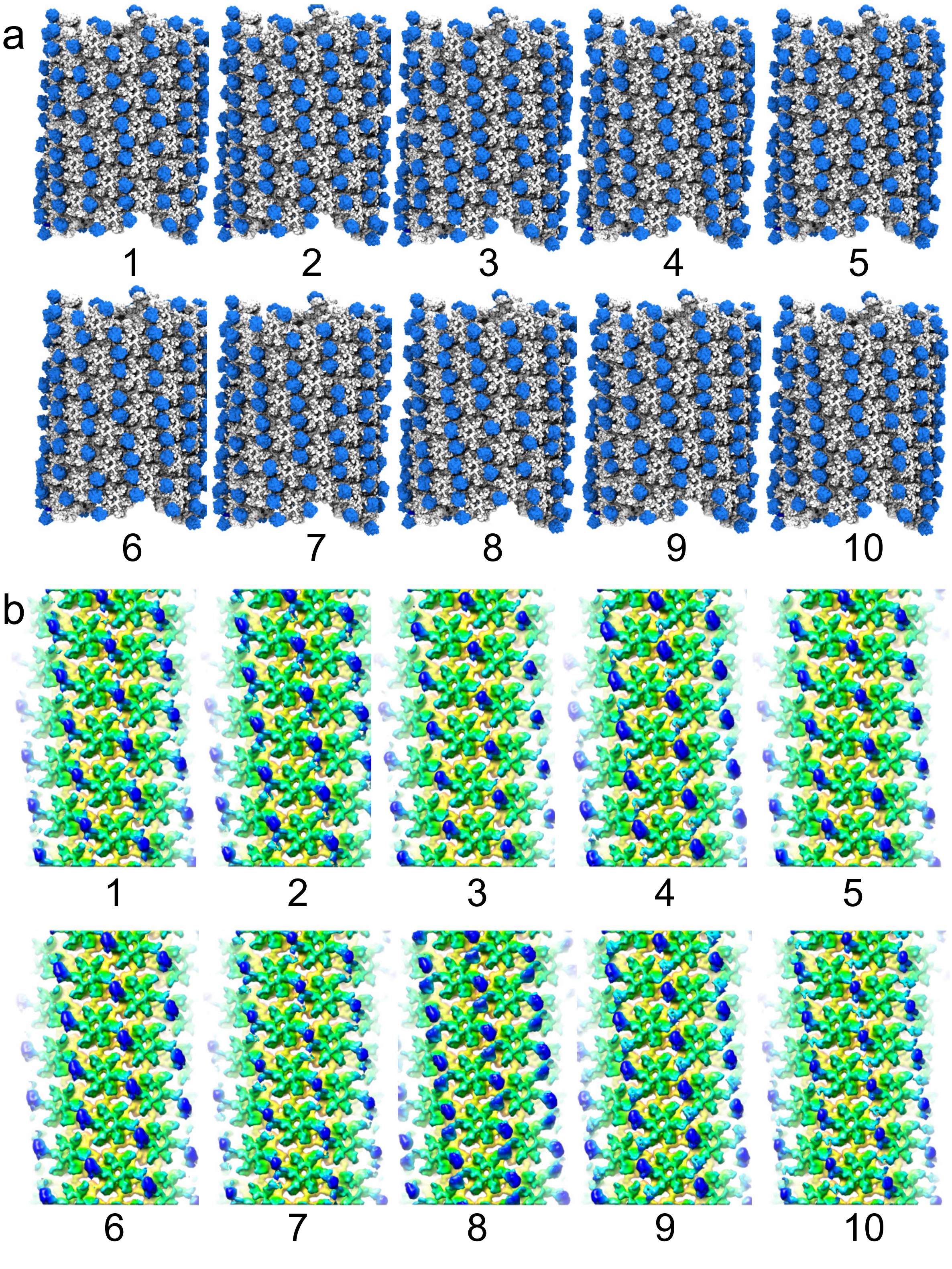

Figure S5. CypA/CA tubular complex models. (a) Ten models (Table S2 - Simulation 2, 100 ns) of random tubular complexes with CA tubes in gray and CypA in blue. (b) Helically symmetrized and simulated density maps of ten models of tubular complexes. All density maps are contoured at 2σ.

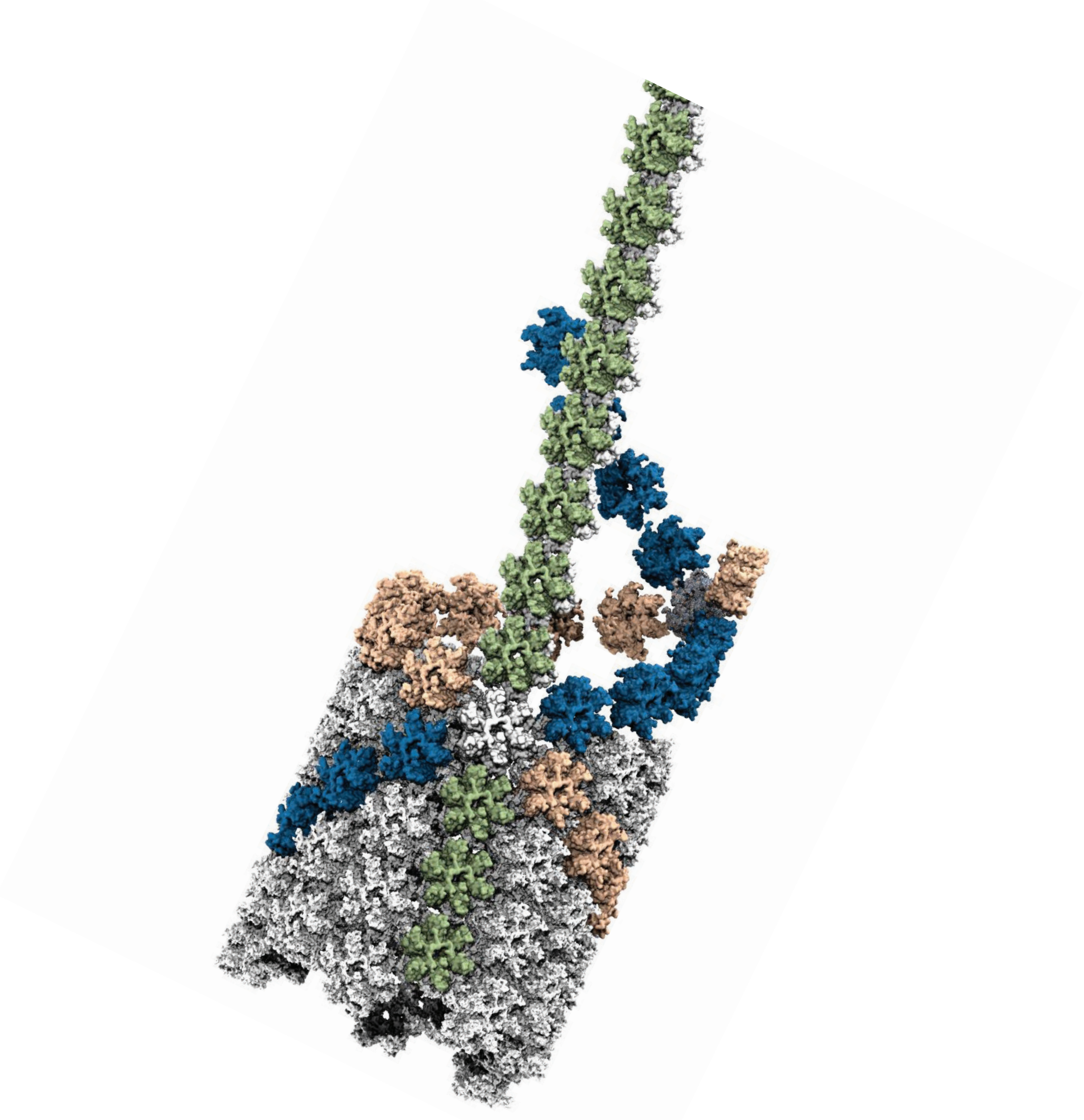

Figure S6. Schematic representation of the three helical directions found in a CypA-CA tube (green, blue and tan). The three helical directions exhibit different curvatures.

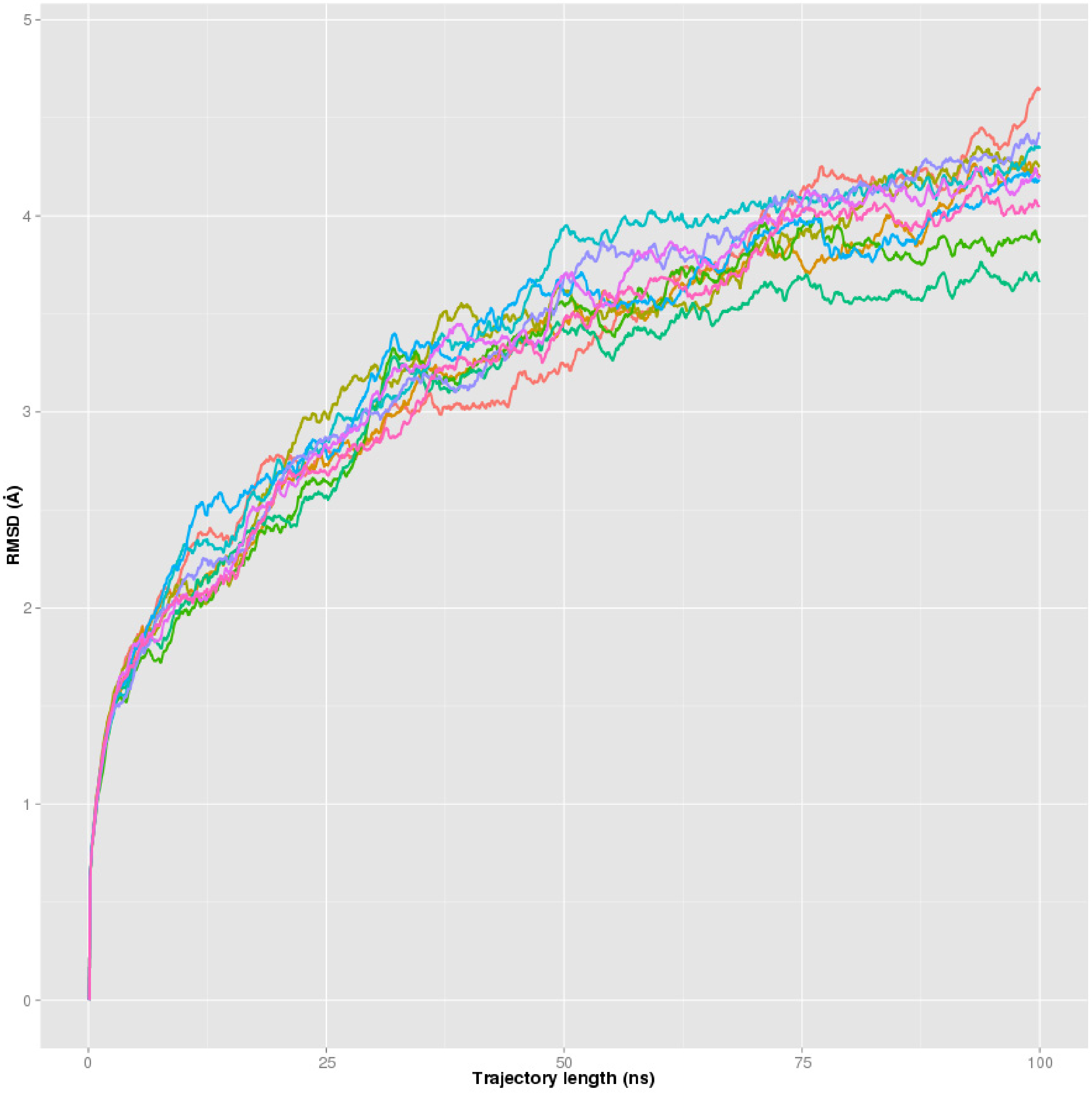

Figure S7. Root mean square deviation of the α-carbon atoms in reference to the initial structure during unrestrained simulations of the MDFF derived model (Table S1 - Simulation 2). Each simulation is represented by a line of different color. The plot shows the convergence of the simulations.

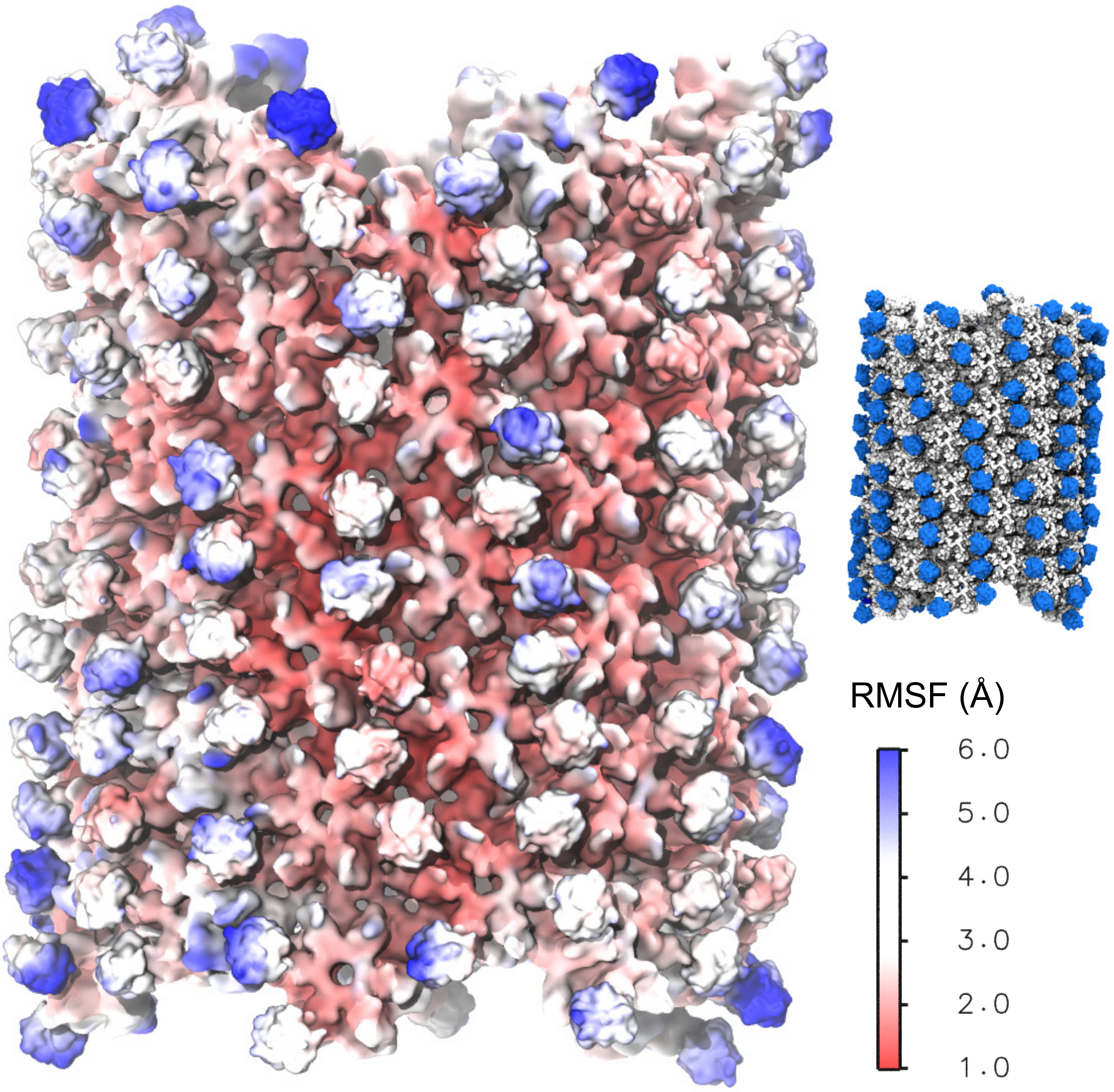

Figure S8. Root mean square fluctuations (RMSF) of the α-carbon atoms in reference to the average structure during unrestrained simulations of the MDFF derived model (Table S1 - Simulation 2). The color scale goes from 1.0 Å to 6.0 Å, with larger fluctuations presented in blue.

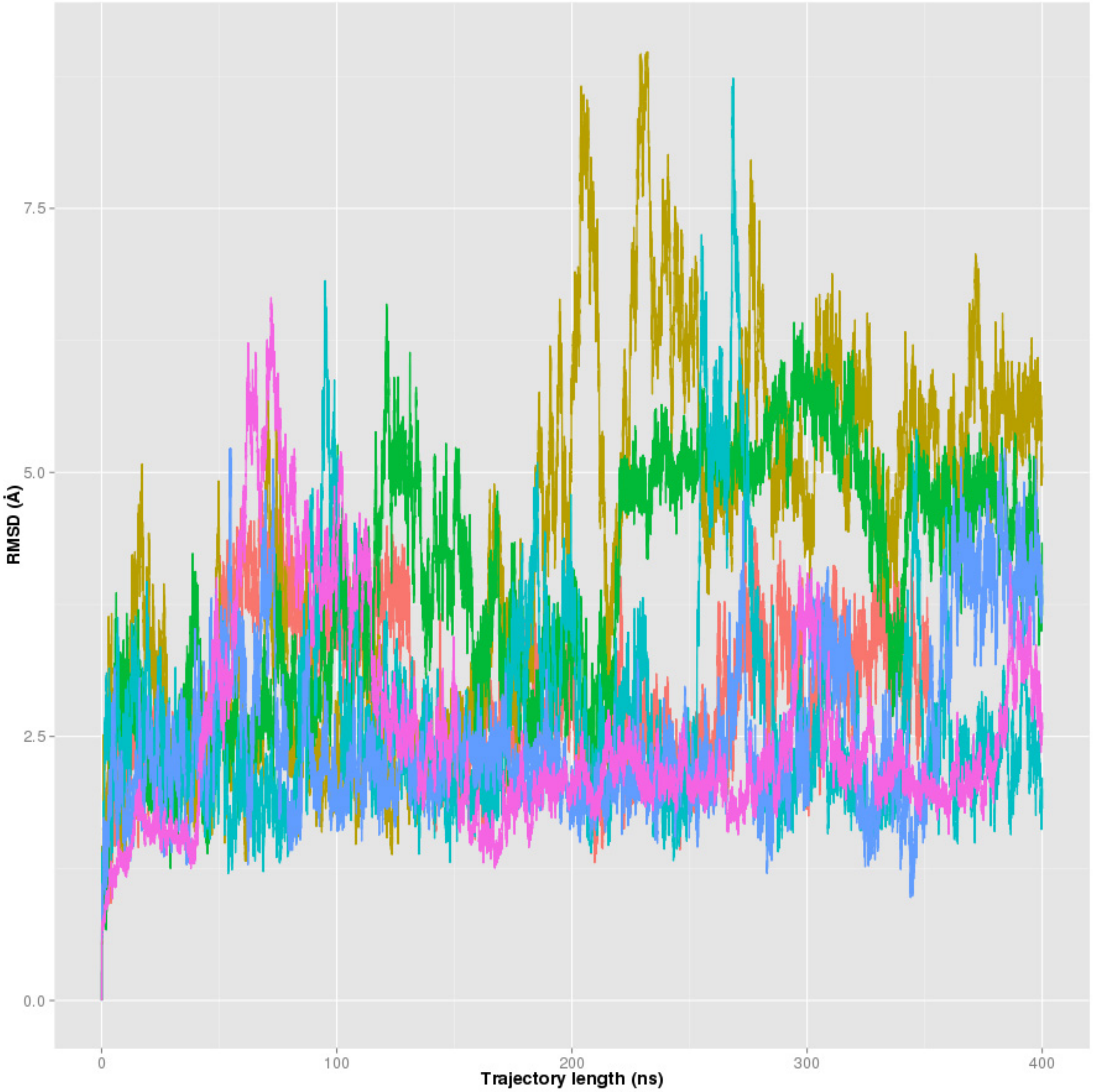

Figure S9. Root mean square deviation of the α-carbon atoms in reference to the initial structure - during unrestrained simulations of the bridge model (Table S1 – Simulation 4). Each simulation is represented by a line of different color. The plot shows the convergence of the simulations during the equilibration phase, corresponding to the first 20ns, and illustrate the global changes observed during production up to 400 ns.

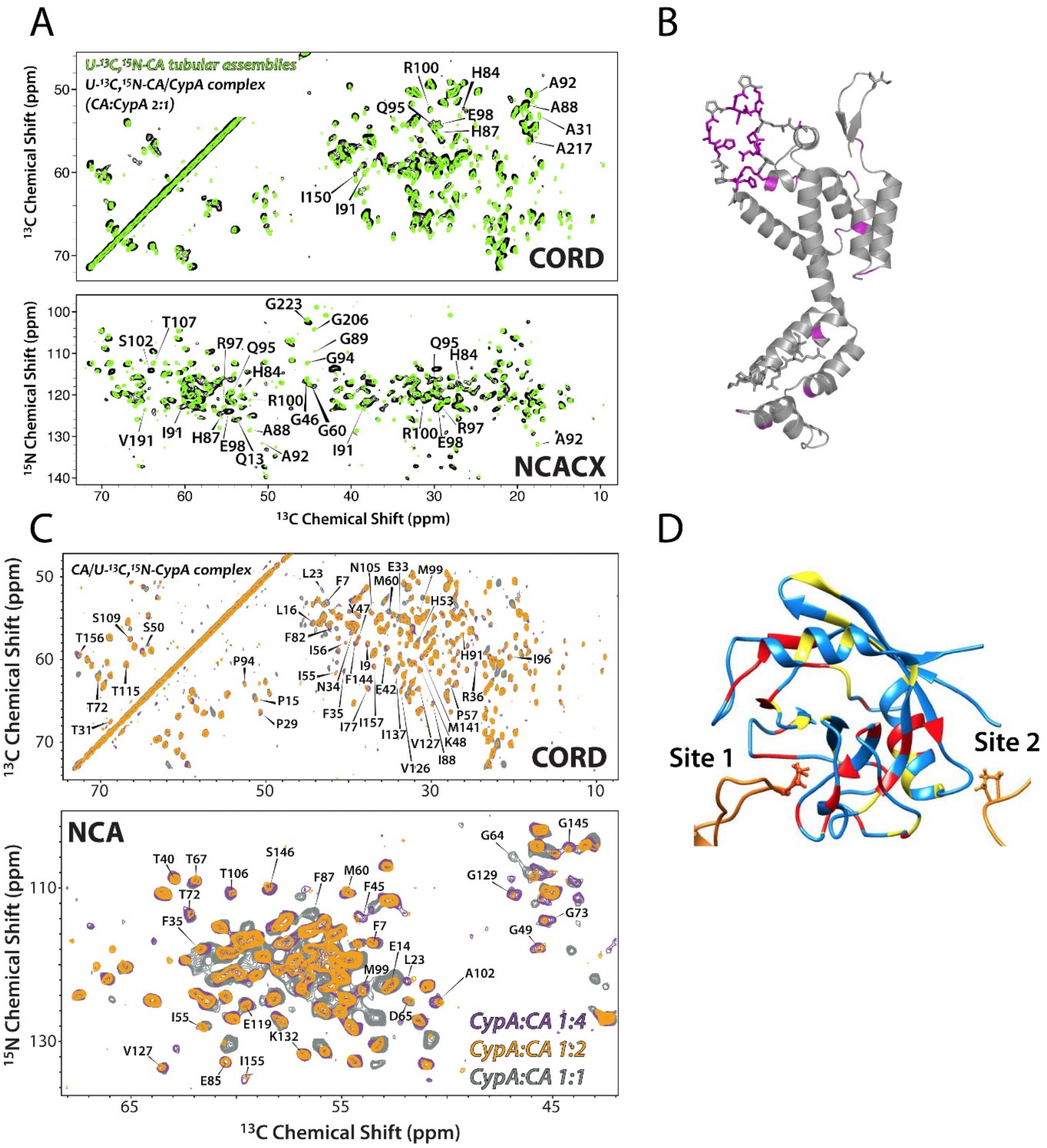

Figure S10. Solid-state MAS NMR studies of the intermolecular interfaces formed between CypA and CA in CypA/CA complex assemblies. A) 2D MAS NMR spectra of U-^{13}C,^{15}N-CA tubular assemblies, free (green) and in complex with CypA (black), acquired at 19.9 T. Top: ^{13}C-^{13}C CORD, bottom: ^{15}N-^{13}C NCACX. The peaks whose chemical shifts or intensities change in the presence of CypA are labeled. B) Mapping of chemical shift and/or intensity changes onto the 3D structure of CA (PDB: 3NTE). Purple indicates residues whose shifts or intensities are perturbed. C) 2D MAS NMR spectra of CA/U-^{13}C,^{15}N-CypA assemblies acquired at 19.9 T. Top: ^{13}C-^{13}C CORD, bottom: ^{15}N-^{13}C NCA. The spectra are shown for the varying CypA:CA ratios: 1:4 (purple), 3:6 (orange), and 6:6 (grey). The peaks whose chemical shifts or intensities change as a function of the CypA:CA ratios are labeled. D) Mapping of chemical shift and/or intensity changes on the 3D structure of CypA. Red and yellow are the changes when the CypA:CA ratio is shifted from 1:4 to 3:6 and 3:6 to 6:6, respectively.

Table S1.. Summary of simulations. Particle count includes atoms corresponding to protein, solvent, and ions. For simulations that employ the Drude polarizable force field, the total particle count also includes lone-pairs and Drude particles. The number of independent repetitions, as well as the length of each simulation is reported in nanoseconds (ns).

Simulation number	Simulation System	Simulation type	System size (Thousands of particles)	Repeats	Length (ns)	Force Field
1	Tubular CypA/CA complex	MDFF	26,500	10	10	Charmm22/ CMAP
2	Tubular CypA/CA complex	MD Production	26,500	10	100	Charmm22/ CMAP
3	CypA/CA bridged	MD Equilibration No restraints	128	7	10	Charmm36
4	CypA/CA bridged	MD Production No restraints	214	7	400	Charmm36/ Drude
5	CypA/CA disjoined	MD Equilibration	98	7	10	Charmm36
6	CypA/CA disjoined	MD Production	187	7	400	Charmm36/ Drude

Table S2. Spectral changes observed in the MAS NMR experiments on CypA-CA assemblies upon changing the CypA:CA ratio.

	CypA:CA- 1:4 to 1:2		CypA:CA- 1:2 to 1:1	
**Residue**	CORD	NCA	CORD	NCA
**F7**			CS	CS
**I9**			ID	
**E14**				II
**P15**			CS	
**L16**			CS, II	
**L23**	ID	CS, ID	ID, D	ID, D
**P29**			ID	
**T31**			CS, ID	
**E33**	II		ID	
**N34**			CS	
**F35**	D	CS		
**R36**	ID		ID	
**T40**				CS
**E42**			II	
**F45**		ID		ID
**Y47**			ID	
**K48**			ID	
**G49**		ID		ID
**S50**	CS			
**H53**	D			
**I55**	II	II	ID	CS
**I56**	CS, II			
**P57**			CS	
**M60**				ID
**G64**		D		II
**D65**		II		II
**T67**		CS		CS
**T72**		ID	CS	II
**G73**		ID, D		ID, D
**I77**	CS		CS, ID	
**F82**	II		II	
**E85**				ID
**F87**		D		II
**I88**			ID	
**H91**	II		ID	
**P94**	CS		ID	
**I96**	CS		ID	
**M99**	II	ID	ID	
**A102**		ID		
**N105**			ID	
**T106**		ID		
**S109**			ID	
**T115**			ID	
**E119**		ID		II
**V126**			CS, ID	
**V127**		ID		CS
**G129**		ID		II
**K132**				ID
**I137**			ID	
**M141**			CS	
**F144**	CS, ID		II	
**G145**		ID		ID
**S146**		ID		
**I155**		ID	CS	ID
**T156**	CS		ID	

ID: peak intensity decrease II: peak intensity increase
D: peak disappearance CS: chemical shift perturbation
